# Patterns of Social Support and LGBTQ + Community Involvement Among Gay, Bisexual, and Other Men Who Have Sex with Men in Australia and Their Effect on HIV-Related Outcomes: A Latent Class Analysis

**DOI:** 10.1007/s10461-025-04632-y

**Published:** 2025-01-27

**Authors:** Curtis Chan, Benjamin R. Bavinton, Horas T. H. Wong, John Rule, Loc Nguyen, Steven Spencer, Martin Holt

**Affiliations:** 1 Kirby Institute, UNSW Sydney, Sydney, Australia; 2 Centre for Social Research in Health, UNSW Sydney, Sydney, Australia; 3 Sydney Nursing School, University of Sydney, Sydney, NSW Australia; 4 National Association of People with HIV Australia, Sydney, NSW Australia; 5 ACON, Sydney, NSW Australia

**Keywords:** Men who have sex with men, HIV, Gay community, Sexual health, Social networks

## Abstract

Peer support from social networks of gay, bisexual, and other men who have sex with men (GBMSM) has been recognised as a critical driver of engagement with HIV prevention. Using data from an online cross-sectional survey of 1,032 GBMSM aged 18 or over in Australia, a latent class analysis was conducted to categorise participants based on social support, LGBTQ + community involvement, and social engagement with gay men and LGBTQ + people. Comparisons between classes were assessed using multivariable multinomial logistic regression. Participants were allocated into four classes: ‘Gay men focused’ (*n* = 293, 28.4%) with high levels of support from gay men, ‘High and diverse’ (*n* = 75, 7.3%) with high support from people with a range of gender and sexual identities, ‘Moderate overall’ (*n* = 177, 17.2%) who reported some support from all sources, and ‘Low overall’ (*n* = 487, 47.2%) who had low support. Lifetime HIV testing was lower in the ‘Low overall’ (78.0%) and ‘Moderate overall’ (81.9%) classes compared to the ‘Gay men focused’ (96.9%) and ‘High and diverse’ (93.3%) groups. Among non-HIV-positive participants (*n* = 971), lifetime PrEP use was lower in the ‘Low overall’ (28.9%) than the ‘Gay men focused’ group (56.2%) but similar in the ‘High and diverse’ (59.7%) and ‘Moderate overall’ (37.5%) groups. Greater levels of social engagement with gay men and LGBTQ + people were associated with higher levels of HIV testing and PrEP use. Interventions are needed to reach GBM who are less engaged with GBMSM networks or LGBTQ + communities.

## Introduction

The success of Australia’s HIV response has been attributed to the strong, ongoing collaboration between members of affected communities, community organisations, governments, clinicians, non-government organisations, and researchers [1, 2]. The significance of the gay community’s contribution to HIV prevention is well documented and includes informing government policy about HIV prevention, becoming advocates and educators in health promotion, and being partners in social research [3–5]. In the context of HIV prevention, ‘gay community’ refers to gay men working together to respond to HIV through grassroots organising, activism and informal peer networks, and also through formally constituted community organisations in which gay men are members of staff [6]. However, we acknowledge that the idea of ‘gay community’ outside of HIV prevention refers to a broader and more ambiguous concept, involving peer networks, socialising, and an imagined collective of gay men and other people who are believed to share common experiences grounded in sexuality [7]. In recent years, community-based organisations in partnership with government health departments and clinical bodies have played a significant role in HIV pre-exposure prophylaxis (PrEP) rollout and implementation [8]. Furthermore, among gay, bisexual, and other men who have sex with men (GBMSM), social engagement with other gay men has been shown to be associated with HIV-related outcomes, such as sexual behaviour, condom use, HIV testing, HIV treatment, and PrEP uptake [9–12]. We acknowledge that the terms ‘gay’, ‘GBMSM’, and ‘LGBTQ+’ are distinct but related concepts, and throughout this article will refer to the specific intended group where appropriate (i.e. ‘gay community’ when talking about gay men, LGBTQ + when talking about a broader collective of people with diverse genders and sexualities).

There are some groups of GBMSM who may not feel connected to ‘gay community’ and may feel actively excluded. For example, bisexual men who experience biphobia or bi-erasure from gay men [13, 14], culturally and linguistically diverse or migrant GBMSM who experience racism and discrimination in Australia [15–17], and increasingly, young GBMSM who experience greater acceptance and less reliance on other GBMSM for social support [18–21]. As many of these groups have lower engagement with sexual health services [22], distance from gay community and less social engagement with gay men may potentially exacerbate health disparities if these groups do not have other sources of support and connection that facilitate health seeking behaviours [22]. Furthermore, these disparities may be reinforced through exclusion from health services due to structural factors such as racism and biphobia [13, 16].

In research, connection to gay community has been variously conceptualised as either an imagined relationship with a community based on sexuality (e.g. “I feel like I am part of the gay community”) or as a set of behaviours and social relationships (e.g. “I go to gay clubs”, “I have gay friends) [3, 23–25]. All of these components are potentially relevant for developing health interventions or health promotion messaging for GBMSM, but the relative importance of each is not clear. Additionally, the historical focus of HIV social and behavioural research on gay men (rather than GBMSM, or LGBTQ + people more generally) reflects the history and politics of the time, but may overlook broader social relations that are important in the sexual health of GBMSM today [1, 3]. For example, it is possible that other forms of social connection (e.g. bisexual men’s relationships with each other, GBMSM’s connections to LGBTQ + people in general) may be associated with similar protective effects for HIV prevention as GBMSM’s connections to gay community and gay men, but these have not been explored in detail before.

Attachment to and engagement with the gay community may be indicative of GBMSM having social support in general (albeit a specific form from gay men), and social support is known to facilitate health seeking behaviours [24–26]. Latent class analysis has been used to categorise LGBTQ + people according to their social network characteristics, showing that those with greater social support from range of sources were more likely to have better mental and physical health outcomes [27, 28]. Applying a similar approach among GBMSM specifically could allow us to understand how broader features of social support (not only social connections with gay men) might impact sexual health by comparing sexual health outcomes between groups of GBMSM with different patterns of social support and connection to LGBTQ + communities and other people. This could help identify groups of GBMSM who may be overlooked in health promotion and interventions that are targeted at GBMSM who are well connected and supported.

This analysis investigated GBMSM’s gay and LGBTQ + community involvement, social support, and social engagement with gay men and other people, and then classified their patterns of social engagement using latent class analysis. The patterns of social engagement identified were then used to assess how different patterns of social connection were related sexual health outcomes. The aim was to assess different patterns of support and connection among GBMSM, and to determine how health behaviours, such as HIV/STI testing and PrEP uptake, varied between different groups of GBMSM with varying levels of connection to gay and LGBTQ + communities and other people.

## Methods

### Participants and Recruitment

The Sexuality, Community Experience and Network Engagement (SCENE) study was an online cross-sectional survey conducted between November 2022 and January 2023 hosted on the survey platform Qualtrics (Provo, UT). Participants were eligible if there were aged 18 or over, identified as male (inclusive of cis and trans men), lived in Australia, and either identified as a gay, bisexual or queer man or had sex with another man in the last five years. Participants were recruited using paid advertising on Facebook, Grindr, and Squirt, and through community-based advertising on Twitter, TikTok, and mailing lists from community organisations. Participants could choose to enter a prize draw to win one of four gift vouchers valued at AUD$100. Responses were checked for duplicate IP addresses, suspicious open text responses, and response duration (outside the interquartile range around the median duration) to ensure response validity. This study received ethics approval from UNSW Human Research Ethics Committee (HC220645).

### Measures

Participants were asked whether they had received social support from 6 groups: gay men, bisexual men, other LGBTQ + people, family members, cisgender heterosexual friends, and romantic partners. Social support in the last year was measured using a 9-item instrument adapted from Frost, Meyer and Schwartz [25]. These included questions such as “In the last year, how many people could you count on to lend you a large sum of money?” and “In the last year, how many people have you spent time with in social activities such as having dinner together, going to the movies, or hanging out?”. For each group, they could respond with “None or N/A”, “One”, “2–5”, “6–10”, or “11 or more”. Participant scores ranged from 0 to 32 for each support group.

Attachment to LGBTQ + community was adapted from the 20-item Gay Community Involvement Index by Foster-Gimbel, Doyle and Engeln [23], replacing “gay community” with “LGBTQ + community”. Participants were asked about their involvement in LGBTQ + community in four subscales: Community activities, nightlife, media, and political activism. Participants were asked how much they agreed with each statement on a 7-point Likert scale from strongly disagree (0) to strongly agree (7). These included items such as “I am a member of a LGBTQ + community group or organisation” and “I watch television programs geared toward the LGBTQ + community”. Each participant received a separate score for each of the four subscales. Community involvement ranged from 0 to 42, nightlife from 0 to 24, media from 0 to 30, and political activism from 0 to 24.

Participants’ social engagement with LGBTQ + people (LGBTQSE) was assessed with two questions: “How many of your friends are LGBTQ+? (“none”, “a few”, “some”, “most”, “all”; scored 0–4) and “How much of your free time is spent with LGBTQ + friends?” (“none”, “a little”, “some”, “a lot”; scored 0–3). The responses were summed to make a scale scored from 0–7. Participants were asked similar questions about social engagement with gay men (GSE) with “LGBTQ+” replaced with “gay men” and scored the same way [9].

Demographics collected included age, sexual identity, highest level of education, employment, and relationship status. Using an established method [29], each participant’s postcode was categorised based on the estimated proportion of male residents who identified as gay. These postcodes were categorised as < 5%, 5–9%, and ≥ 10% gay residents. Participants’ HIV status was assessed by asking about the result of their last HIV test (HIV-positive, HIV-negative, Don’t know/Prefer not to say). Participants who had never received an HIV test were combined with those who did not know their status or preferred not to say (into an untested/unknown category).

Participants were asked when they had their last HIV test. Responses for all participants were dichotomised based on whether they had ever been tested for HIV or not, and responses for participants not living with HIV were dichotomised into two groups (tested for HIV in the last twelve months or not). Among the whole sample, comprehensive STI testing was assessed by asking participants whether they had received an anal swab, throat swab, urine sample, or blood test for syphilis in the last twelve months. Participants were considered to have had comprehensive STI testing if they reported receiving all four tests [30]. Participants self-reported whether they received any STI diagnosis in the last twelve months. Among those who were not living with HIV, participants were dichotomised into two groups (having ever taken PrEP or not). Among participants who reported casual sex, participants were categorised into three HIV prevention coverage groups: no condomless sex during anal or vaginal sex in the last six months (i.e. consistent condom use or no anal/vaginal sex), any condomless sex but the participant was taking PrEP or had a UVL, or condomless sex without PrEP or UVL. The first two groups were classified as safe sex (no risk of HIV transmission), the third group as presenting a risk of HIV transmission [31].

### Analyses

A latent class analysis was conducted by entering measures of sources of social support, LGBTQ + community involvement index, and social engagement with gay men and LGBTQ + people into the latent class model (Table 1). To determine the ideal number of classes for the analysis, several model fit indicators were used for models between two and seven classes (Table 2). These included Akaike Information Criterion (AIC), Bayesian Information Criterion (BIC), and model entropy. Lower values of AIC, BIC, and log-likelihood indicated better model fit, and entropy values closer to 1 indicated better separation between classes. Each class was required to have more than 5% of the total sample. Each participant was then assigned to the class for which they had the highest probability of class membership.

**Table 1:** Social support, LGBTQ + community involvement, social engagement with gay men, and social engagement with LGBTQ + by class

	Gay men focused	High and diverse	Moderate overall	Low overall
Source of social support (0–32) from:				
Gay male friends	11.23 (4.31)	18.36 (4.75)	8.42 (4.32)	3.85 (3.60)
Bi + male friends	2.50 (3.62)	11.90 (6.71)	5.56 (4.39)	1.76 (3.01)
Other LGBTQ + friends	2.35 (2.97)	14.79 (5.19)	9.67 (4.23)	1.51 (2.50)
Cisgender heterosexual friends	5.27 (5.62)	11.86 (7.83)	10.44 (6.86)	5.30 (6.15)
Family	9.74 (5.16)	13.49 (7.25)	12.40 (5.12)	9.05 (5.57)
LGBTQ + community involvement index				
Community (0–24)	12.42 (9.35)	21.33 (10.19)	13.72 (9.27)	5.88 (7.20)
Media (0–30)	17.79 (6.45)	21.22 (5.78)	19.74 (5.76)	13.01 (7.21)
Nightlife (0–24)	14.12 (5.94)	16.88 (5.66)	13.76 (5.04)	6.86 (5.59)
Political activism (0–24)	8.12 (5.75)	13.61 (5.48)	10.21 (6.08)	4.78 (4.58)
Social engagement (0–7)				
Gay social engagement	4.60 (1.01)	4.73 (1.18)	2.46 (0.83)	1.84 (0.93)
LGBTQ + social engagement	4.53 (1.01)	5.04 (0.99)	3.60 (1.15)	1.99 (1.04)
Total	293	75	177	487

**Table 2:** Model fit statistics for 2–7 classes

Classes	AIC	BIC	Entropy
2	65852.53	66030.34	0.8303
3	65208.71	65450.74	0.7494
**4**	**64783.24**	**65084.53**	**0.6731**
5	64598.46	64968.9	0.6171
6	64298.93	64733.58	0.6213
7	64093.70	64592.56	0.6072

The demographic and behavioural characteristics of the different classes were compared using chi-square tests for categorical measures and analysis of variance (ANOVA) tests for continuous variables. Several multivariable multinomial logistic regression models were conducted to compare classes on HIV and sexual health-related outcome variables. These outcome variables included having ever tested for HIV, receiving comprehensive STI testing in the last year, and any STI diagnosis in the last 12 months (with models created for all participants). Models for HIV testing in the last year, the number of HIV tests in the last year, and ever having taken PrEP were restricted to participants not living with HIV. An analysis of HIV prevention coverage was restricted to participants who reported casual sex in the last six months. Multiple imputation by chained equations (MICE) with 100 iterations was conducted to impute missing data on demographic and sexual behaviour variables. Models were adjusted for potential confounders including age, education, employment, postcode, country of birth, relationship status, and self-reported number of sexual partners. Analyses were conducted in Stata (version 18, StataCorp, College Station, USA).

## Results

There were 1,507 participants who consented to the survey. Of these, 15 were ineligible (lived outside of Australia or aged under 18) and were excluded. Of the remaining 1,492, an additional 26 responses were considered invalid due to duplicate IP addresses or suspicious response patterns. From the remaining sample, 1,032 participants progressed to the final page of survey. Further analyses are restricted to these 1,032 participants. Data was imputed for 49 missing responses in total, and between 1 and 32 responses were imputed on any single variable. The mean age was 41.8 years (SD = 13.5), 645 (62.5%) identified as gay, 299 (29.0%) identified as bisexual, and 88 (8.5%) identified as a different sexual identity. There were 174 (16.9%) participants who were born in a country other than Australia, 822 (79.7%) identified their cultural background/ethnicity as Australian, 194 (23.6% of 822) identified with another cultural background/ethnicity, and 115 (11.1%) spoke a language other than English at home.

### Latent Classes

AIC, BIC and entropy were lowest for a seven class model (Table 1). However, models with five, six, and seven classes produced multiple classes with small sample sizes. Considering sample size, higher entropy, and model interpretability, a model with four classes was retained. Table 2 shows the mean score on each scale entered into the model for each of the four classes.

Class 1 (*n* = 293, 28.4%) was labelled ‘Gay men focused’ – participants in this class indicated strong social support from gay men, but low support from most other groups. This group had moderate involvement in LGBTQ + community, and were very socially engaged with gay men and LGBTQ + people. This is the only group who were more socially engaged with gay men than with LGBTQ + people generally. Class 2 (*n* = 75, 7.3%) was labelled ‘High and diverse’ – they reported strong social support from a variety of sources, high involvement with LGBTQ + community, and were the most socially engaged with gay men and LGBTQ + people of all the classes. Class 3 (*n* = 177, 17.2%) was labelled ‘Moderate overall’ – they received moderate social support from a range of groups, had some involvement in LGBTQ + community, and had some social engagement with gay men and LGBTQ + people. Class 4 (*n* = 487, 47.2%) was labelled ‘Low overall’ – this group reported low social support from most groups with the highest support from family, low involvement in the LGBTQ + community, and the lowest social engagement with both gay men and LGBTQ + people.

### Demographic and Behavioural Characteristics and Differences Between Classes

The demographic characteristics of each class are shown in Table 3. The ‘Moderate overall’ class was the youngest (Mean age = 35.3, SD = 13.3), followed by the ‘High and diverse’ (M = 37.6, SD = 11.4), ‘Gay men focused’ (M = 43.4, SD = 12.3), and ‘Low overall’ (M = 43.8, SD = 13.6) classes. Those in the ‘Moderate overall’ and ‘Low overall’ classes were less likely to identify as gay (55.4% and 49.5% respectively) compared to the ‘Gay men focused’ and ‘High and diverse’ groups (85.7% and 77.3% respectively). Conversely, the ‘Moderate overall’ and ‘Low overall’ classes were more likely to identify as bisexual (31.6% and 39.8% respectively) compared to the ‘Gay men focused’ and ‘High and diverse’ groups (10.9% and 22.7% respectively). The ‘Gay men focused’ class was the most likely to live in a postcode with a ≥ 10% gay male population (14.7%) compared to the ‘High and diverse’ (9.9%), ‘Moderate overall’ (5.7%) and ‘Low overall’ (3.6%) classes. Those in the ‘Gay men focused’ and ‘High and diverse’ classes were slightly more likely to be university educated (45.4% and 48.0% respectively) compared to both the ‘Moderate overall’ (37.3%) and ‘Low overall’ (36.4%) classes. There were no statistically significant differences in employment or relationship status across classes.

**Table 3:** Demographic and behavioural characteristics of classes

	Gay men focused	High and diverse	Moderate overall	Low overall	*p*
Age (M/SD)	43.4 (12.3)	37.6 (11.4)	35.3 (13.3)	43.8 (13.6)	< 0.001
Sexual identity					< 0.001
Gay	251 (85.7)	55 (73.3)	98 (55.4)	241 (49.5)	
Bisexual	32 (10.9)	17 (22.7)	56 (31.6)	194 (39.8)	
Other	10 (3.4)	3 (4.0)	23 (13.0)	52 (10.7)	
Gay postcode					< 0.001
≥ 10%	42 (14.7)	7 (9.9)	10 (5.7)	17 (3.6)	
5–9%	37 (13.0)	16 (22.5)	11 (6.3)	34 (7.2)	
< 5%	206 (72.3)	48 (67.6)	153 (87.9)	419 (89.1)	
Born outside of Australia	65 (22.2)	15 (20.0)	15 (8.5)	65 (22.2)	0.001
University education	133 (45.4)	36 (48.0)	66 (37.3)	177 (36.4)	0.034
Full time employment	159 (54.3)	43 (57.3)	85 (48.0)	277 (57.0)	0.217
In romantic relationship	130 (44.4)	32 (42.7)	73 (41.2)	200 (41.2)	0.836
HIV status					< 0.001
HIV-positive	28 (9.6)	8 (10.7)	9 (5.1)	16 (3.3)	
HIV-negative	243 (82.9)	62 (82.7)	133 (75.1)	358 (73.5)	
Don’t know/prefer not to say/untested	22 (7.5)	5 (6.7)	35 (19.8)	113 (23.2)	
No. men sex partners last 6 months (M/SD)	12.02 (16.97)	24.75 (52.33)	8.16 (15.43)	6.45 (11.68)	< 0.001
No. women sex partners last 6 months (M/SD)	0.16 (1.07)	0.92 (3.74)	0.68 (2.56)	0.59 (1.37)	< 0.001
No. non-binary sex partners last 6 months (M/SD)	0.19 (0.71)	1.89 (5.73)	0.27 (0.84)	0.18 (1.09)	< 0.001
**Total**	**293**	**75**	**177**	**487**	

Chi square for categorical, ANOVA for continuous

The ‘Moderate overall’ and ‘Low overall’ classes were more likely to have an unknown HIV status (19.8% and 23.2% respectively) compared to the ‘Gay men focused’ and ‘High and diverse’ classes (7.5% and 6.7% respectively). Conversely, there were higher proportions of people living with HIV in the ‘Gay men focused’ (9.6%) and ‘High and diverse’ (10.7%) classes. The ‘High and diverse’ class had the highest mean number of male, female, and non-binary sexual partners in the last 6 months. The ‘Gay men focused’ class had the next highest mean number of male sexual partners, followed by the ‘Moderate overall’ and ‘Low overall’ groups. However, the ‘Gay men focused’ class had fewer female and non-binary partners compared to the ‘Moderate overall’ class, and fewer female partners compared to the ‘Low overall’ class.

### HIV and Sexual Health Outcomes

For the multivariable multinomial logistic regression models, the ‘Gay men focused’ class was selected as the reference group (Table 4). Compared to the proportion of the ‘Gay men focused’ class who had ever been tested for HIV (96.9%), the ‘Moderate overall’ class (81.9%) was significantly less likely to have ever tested for HIV (aRRR = 0.32, 95%CI = 0.13–0.74, *p* = 0.008) as was the ‘Low overall’ class (78.0%, aRRR = 0.16, 95%CI = 0.07–0.34, *p* < 0.001). The proportion who had ever tested for HIV in the ‘High and diverse’ class (93.3%) was not significantly different (aRRR = 0.45, 95%CI = 0.13–1.51, *p* = 0.197). Among those not living with HIV (*n* = 971), HIV testing in the last year was similar to the pattern observed for lifetime HIV testing, with the ‘Moderate overall’ class (58.3%; aRRR = 0.63, 95%CI = 0.39–0.99, *p* = 0.050) and the ‘Low overall’ class (51.4%; aRRR = 0.46, 95%CI = 0.31–0.66, *p* < 0.001) being less likely to have recently tested compared to the ‘Gay men focused’ class (75.5%). The ‘Gay men focused’ and ‘High and diverse’ classes had similar levels of recent testing (77.6%; aRRR = 0.83, 95%CI = 0.41–1.67, *p* = 0.605).

**Table 4:** Multinomial logistic regression models on sexual health outcome measures

	Gay men focused *n* (%)	High and diverse *n* (%)	Moderate overall *n* (%)	Low overall *n* (%)	High and diverse vs. Gay men focused (aRRR)	Moderate overall vs. Gay men focused (aRRR)	Low overall vs. Gay men focused (aRRR)
*All participants*							
Ever tested for HIV	284 (96.9)	70 (93.3)	145 (81.9)	380 (78.0)	0.45 (0.13–1.51)	0.32 (0.13–0.74)**	0.16 (0.07–0.34)***
Had all STI tests in the last 12 months	250 (85.3)	65 (86.7)	132 (74.6)	349 (71.7)	1.28 (0.70–2.34)	0.83 (0.54–1.30)	0.57 (0.40–0.81)**
Any STI in last 12 months	105 (35.8)	26 (34.7)	46 (26.0)	114 (23.4)	0.55 (0.27–1.12)	0.69 (0.41–1.17)	0.60 (0.40–0.90)*
**Total**	**293**	**75**	**177**	**487**			
*Participants not living with HIV*							
Tested for HIV in last year	200 (75.5)	52 (77.6)	98 (58.3)	242 (51.4)	0.83 (0.41–1.67)	0.60 (0.39–0.99)*	0.46 (0.31–0.66)***
Have ever taken PrEP	149 (56.2)	40 (59.7)	63 (37.5)	136 (28.9)	0.98 (0.52–1.86)	0.76 (0.48–1.21)	0.50 (0.35–0.71)***
**Total**	**265**	**67**	**168**	**471**			
*Participants reporting casual sex*							
Prevention coverage							
No condomless sex	29 (12.9)	6 (10.2)	29 (23.6)	77 (23.3)	0.82 (0.27–2.53)	1.89 (0.95–3.74)	1.82 (1.04–3.16)*
Condomless sex with PrEP or UVL	116 (51.6)	32 (54.2)	36 (29.3)	81 (24.6)	1.13 (0.53–2.38)	0.86 (0.48–1.54)	0.60 (0.38–0.94)*
Condomless sex without PrEP or UVL	80 (35.6)	21 (35.6)	59 (47.2)	172 (52.1)	**REF**	**REF**	**REF**
**Total**	**252**	**59**	**123**	**330**			

“Gay men focused” class is the reference group. Models are adjusted for age, education, employment, postcode, country of birth, relationship status, number of male partners, number of female partners, and number of non-binary partners

*p < 0.05

**p < 0.01

***p < 0.001

In the whole sample, those in the ‘Low overall’ class were less likely to have received comprehensive STI testing in the last year (51.4%) compared to the ‘Gay men focused’ class (85.3%; aRRR = 0.57, 95%CI = 0.40–0.81, *p* = 0.002), while the ‘High and diverse’ (86.7%; aRRR = 1.28, 95%CI = 0.70–2.33, *p* = 0.417) and ‘Moderate overall’ classes (74.6%; aRRR = 0.83, 95%CI = 0.54–1.30, *p* = 0.421) had similar levels of comprehensive testing to the ‘Gay men focused’ class. This pattern was also observed for any STI diagnoses in the last 12 months with the ‘Low overall’ class being less likely to report a STI diagnosis (23.4%; aRRR = 0.60, 95%CI = 0.40–0.90, *p* = 0.015) than the ‘Gay men focused’ (35.8%) class.

Among participants not living with HIV (*n* = 971), the ‘Low overall’ class was significantly less likely to have ever taken PrEP (28.9%; aRRR = 0.50, 95%CI = 0.35–0.71, *p* < 0.001) compared to the ‘Gay men focused’ classes (56.2%), with no statistically significant differences with the ‘High and diverse’ (59.7%) and ‘Moderate overall’ (37.5%) classes.

Among participants who reported casual sex (inclusive of all HIV statuses) (*n* = 737), compared to having condomless sex without PrEP or UVL, the ‘Low overall’ class was more likely than the ‘Gay men focused’ class to not have condomless sex (23.3% vs. 12.9%, aRRR = 1.81, 95%CI = 1.04–3.16, *p* = 0.035), and less likely to report having condomless sex with PrEP or UVL (24.6% vs. 51.6%, aRRR = 0.60, 95%CI = 0.38 = 0.94, *p* = 0.027). There were no significant differences between the ‘Gay men focused’, ‘Moderate overall’ and ‘High and diverse’ classes on prevention coverage. Among those reporting condomless sex without PrEP or UVL (*n* = 332), 24.1% were from the ‘Gay men focused’ class, 6.3% from the ‘High and diverse’ class, 17.8% from the ‘Moderate overall’ class, and 52.0% from the ‘Low overall’ class.

## Discussion

This study sought to investigate how connection to LGBTQ + community, social support from a range of sources, and social engagement with gay men and LGBTQ + people affected sexual health outcomes among GBMSM. From our latent class analysis, four classes emerged with different sources of support, and varying levels of involvement and social engagement with LGBTQ + community. We found that those with low or moderate social support from a range of sources were less likely to test for HIV/STIs and take PrEP compared to those who received support mainly from gay men or those who received support from a wider range of people. These findings are consistent with previous work showing that social support and involvement in gay community may be important for sexual health [23]. However, importantly we have also shown that GBMSM may receive social support from a broader range of sources than just gay men (particularly other LGBTQ + people), and support from these sources may also be related to positive health outcomes. By demonstrating that subgroups of GBMSM have several different patterns of social support and engagement with LGBTQ + community, our results suggest useful avenues for health and community organisations to improve their interventions and initiatives to better reach and engage GBMSM who continue to be underserviced.

The ‘Gay men focused’ group represented over a quarter of the sample, primarily found support from gay men, and was well engaged with sexual health services. This group was the most likely to identify as gay men, had nearly all been tested for HIV, with a relatively high proportion recently testing for HIV and STIs and having PrEP experience. The characteristics of this group largely aligned with the highly community and service engaged GBMSM that have been well described in previous research in Australia [2, 32]. This group is the one that previous research has suggested is most engaged in HIV testing, treatment, and PrEP [9–11, 33, 34]. Comparing the ‘Gay men focused’ class to both the ‘High and diverse’ and ‘Moderate overall’ classes demonstrate that support specifically from gay men appears to be an important common factor for many GBMSM and is associated with beneficial sexual health outcomes. For example, those in the ‘High and diverse’ class received substantial social support from gay men and had similar levels of HIV/STI testing and PrEP experience to the ‘Gay men focused’ class. However, we acknowledge that those in the ‘High and diverse’ class also had support from other people (including LGBTQ + people) which may have fostered beneficial sexual health outcomes.

It is possible that GBMSM with a strong network of other gay men may engage in protective behaviours because they are more likely to be well informed and engaged with HIV testing, treatment, and PrEP. Potential underlying mechanisms that explain how connection to gay men and gay/LGBTQ + community could encourage protective behaviours include sharing peer-based norms and modelling (observing others’ behaviour) within a network [35, 36], access to informal sexual health information such as sharing where to go for HIV/STI testing [33, 37], and greater exposure to health promotion and social marketing for sexual health services targeting GBMSM at LGBTQ + community venues and events [38, 39]. One interpretation of our findings is that HIV prevention interventions have been effective for GBMSM who are well supported by and highly engaged with other gay men and LGBTQ + community. Yet this means that those who are not well connected to gay men or LGBTQ + community may remain underserved in HIV prevention efforts, targeted interventions, and peer education. Since many participants derived support from a range of people other than gay men, further investigation about how networks beyond gay men could be engaged in health promotion and interventions for HIV prevention should be considered.

The largest group of participants were those who had relatively lower levels of social support from most sources and were not highly involved with LGBTQ + community. The ‘Low overall’ group, representing nearly half of the sample, were characterised by having comparatively low social support from friends regardless of gender or sexual identity, and their family members were their main source of support. This group was less engaged with sexual health services compared to other groups, even when controlling for number of sexual partners, and represented over half of participants who had condomless sex without PrEP or UVL. This is consistent with evidence that greater social support and community connectedness are associated with sexual health-seeking behaviour [23, 26, 33, 40]. More broadly, previous work has argued that social support in general is important for health-seeking behaviour beyond sexual health [41].

As GBMSM and LGBTQ + individuals are at a higher risk of social isolation due to rejection from friends and family [42, 43], having less social support and involvement in community groups is likely to have an impact on health behaviours such as being more affected by stressors and having less peer-based guidance about health-seeking behaviours [41]. Despite family members being the main source of social support in this group, the ‘Low overall’ group still had the lowest average support from family compared to the other three groups. Similarly, while support from cisgender heterosexual friends was the next highest source of social support, this was lower than the ‘High and diverse’ and ‘Moderate overall’ groups, and was similar to the ‘Gay men focused’ group. While those in the ‘Low overall’ group were generally less sexually active, within this class more than a fifth (22.0%) had never had an HIV test and 52.1% of those reporting casual sex reported having condomless anal sex without PrEP or UVL. Engaging with this group in health promotion and targeted interventions for any health behaviour is likely to be challenging if relying on peer-based social networks, and may be further complicated due to stigma and discomfort associated with sexuality and HIV [44, 45].

There were a sizeable proportion of bisexual men in the ‘Low overall’ and ‘Moderate overall’ classes. These classes had comparatively lower levels of social support and involvement in LGBTQ + community, were generally less sexually active (though more likely to have female sexual partners), and significantly less likely to engage with HIV/STI testing compared to the ‘Gay men focused’ class. A higher proportion of bisexual men in these groups could be indicative of bisexual men finding support beyond gay and LGBTQ + communities [46] and potential biphobia or bi-erasure from LGBTQ + communities [13, 46]. Interestingly, while the ’Moderate overall’ class received more support from bisexual men (M = 5.56, SD = 4.39) compared to the ‘Gay men focused’ class (M = 2.50, SD = 3.62), within the ‘Moderate overall’ class, they received less support from bisexual men compared to gay men (M = 8.42, SD = 4.32), other LGBTQ + friends (M = 9.67, SD = 4.23), and cisgender heterosexual friends (M = 10.44, SD = 6.86). Similarly, participants in the ‘Low overall’ class were also more likely to identify as bisexual but still received less social support from bisexual men compared to gay men, and relied more on family and cisgender heterosexual friends. As bisexual men are likely to engage with gay and LGBTQ + community differently than gay men [47], further research is needed to understand how the sexual health needs of bisexual men are distinct from gay men, and can be met in culturally appropriate ways. Additionally, future health promotion and targeted interventions should explicitly include bisexual men as a separate group rather than uncritically grouping them together with gay men.

There are several implications of these findings. There are still gaps in health promotion and targeted interventions for GBMSM who may not be well connected to GBMSM networks and LGBTQ + community. Future health promotion and research should aim to include these less connected groups of GBMSM while still maintaining engagement with those who are well connected. Particularly, there is room for improvement in sexual health outcomes among those with lower levels of social support, LGBTQ + community involvement, and social engagement with gay men and LGBTQ + people. While the LGBTQ + community involvement was low among the ‘Low overall’ group, in this class there was a relatively high score on the ‘Media’ subscale of the LGBTQ + Community Involvement Index. LGBTQ + media could be a potential avenue for health promotion for the ‘Low overall’ group. Furthermore, there are still potential gains to be made among the ‘Moderate overall’ group. Interventions targeting either those with low overall support or those with high support from gay men are likely to reach some of those with moderate overall support. We found considerable support from family and cisgender heterosexual friends in the ‘Low overall’ and ‘Moderate overall’ classes compared to other sources of social support. While interventions utilising GBMSM networks for sexual health are well researched, there is less research in Australia on interventions and health promotion for sexual health that involve other people beyond LGBTQ + networks. More research may be needed to develop innovative strategies for promoting HIV/STI testing, PrEP, and treatment to those with less social support from LGBTQ + people and involvement with LGBTQ + community.

There are several limitations to this analysis. As a cross-sectional survey, it is not possible to determine causal relationships between social support and access to sexual health services. We did not capture information about overall network size (i.e. the number of people in each group). Questions concerning social support adapted from Frost, Meyer and Schwartz [25] asked about support received in the last year (e.g. as “In the last year, how many people could you count on to lend you a large sum of money?”). Individuals could feel supported by their networks but not have needed specific support in the last year, such as needing a large sum of money, and therefore these measures may have underestimated their levels of support. The survey did not capture information about the quality of relationships and support with friends and family, and future work could investigate this in more detail. As most participants were recruited from dating and hookup apps and websites such as Grindr and Squirt, and the survey was only available in English, the sample is likely to be more sexually active and not necessarily representative of all GBMSM. Those born overseas and identifying with other cultural or linguistic backgrounds were underrepresented in our sample. As this group is a priority population in HIV epidemiology in Australia and more likely to experience structural barriers to connection to gay and LGBTQ + communities due to racism, more evidence is needed to understand how social support and connection to communities affect HIV-related outcomes in this population. As this analysis focused on GBMSM, we do not know whether levels of support and community involvement are comparable to that of the general population or other LGBTQ + groups.

## Conclusion

Our results show that there are a substantial proportion of GBMSM who are not well supported by other LGBTQ + people or GBMSM. GBMSM who are well connected to gay men and LGBTQ + community are more engaged with sexual health services compared to those with moderate or low connection, which may be the result of targeted interventions and health promotion for HIV prevention among gay and LGBTQ + networks. We found gaps in sexual health outcomes among those who were less involved with gay men and LGBTQ + community. Health promotion and interventions need to recognise the different social support and engagement patterns of different groups of GBMSM. To replicate the success achieved among GBMSM who are highly connected to each other may require innovation in groups who are less well connected to GBMSM or LGBTQ + networks and communities.

## Data Availability

Data can be supplied upon reasonable request to the corresponding author.
